# Correction: Early mortality in tuberculosis patients initially lost to follow up following diagnosis in provincial hospitals and primary health care facilities in Western Cape, South Africa

**DOI:** 10.1371/journal.pone.0300768

**Published:** 2024-03-14

**Authors:** Muhammad Osman, Sue-Ann Meehan, Arne von Delft, Karen Du Preez, Rory Dunbar, Florian M. Marx, Andrew Boulle, Alex Welte, Pren Naidoo, Anneke C. Hesseling

There are errors in the Author Contributions. The correct contributions are:

**Conceptualization:** Muhammad Osman, Sue-Ann Meehan, Karen Du Preez, Florian M. Marx, Alex Welte, Pren Naidoo, Anneke C. Hesseling.

**Data curation:** Muhammad Osman, Sue-Ann Meehan, Arne von Delft, Rory Dunbar, Andrew Boulle.

**Formal analysis:** Muhammad Osman, Sue-Ann Meehan, Arne von Delft, Florian M. Marx, Andrew Boulle, Alex Welte, Pren Naidoo, Anneke C. Hesseling.

**Funding acquisition:** Muhammad Osman, Sue-Ann Meehan, Anneke C. Hesseling.

**Investigation:** Muhammad Osman, Sue-Ann Meehan, Arne von Delft.

**Methodology:** Muhammad Osman, Sue-Ann Meehan, Arne von Delft, Karen Du Preez, Rory Dunbar, Florian M. Marx, Andrew Boulle, Alex Welte, Pren Naidoo, Anneke C. Hesseling.

**Project administration:** Muhammad Osman, Sue-Ann Meehan.

**Resources:** Anneke C. Hesseling, Andrew Boulle.

**Supervision:** Andrew Boulle, Alex Welte, Pren Naidoo, Anneke C. Hesseling.

**Validation:** Muhammad Osman, Sue-Ann Meehan, Arne von Delft.

**Writing–original draft:** Muhammad Osman.

**Writing–review & editing:** Muhammad Osman, Sue-Ann Meehan, Arne von Delft, Karen Du Preez, Rory Dunbar, Florian M. Marx, Andrew Boulle, Alex Welte, Pren Naidoo, Anneke C. Hesseling.
