# Supplementary material for: Early mortality in tuberculosis patients initially lost to follow up following diagnosis in provincial hospitals and primary health care facilities in Western Cape, South Africa
Source: PLoS One. 2021 Jun 14;16(6):e0252084. doi: 10.1371/journal.pone.0252084 (PMC8202951; doi:10.1371/journal.pone.0252084)
Supplement: S1 File — (PDF) [file pone.0252084.s002.pdf]

Supplementary material

Early mortality in tuberculosis patients initially lost to follow up following diagnosis in provincial hospitals and primary health care facilities in Western Cape, South Africa

Muhammad Osman[1], Sue-Ann Meehan[1], Arne von Delft[2,3], Karen Du Preez[1], Rory Dunbar[1], Florian M Marx[1,4], Andrew Boulle[2,3], Alex Welte[4], Pren Naidoo[1], Anneke C Hesseling[1]

1. Desmond Tutu TB Centre, Department of Paediatrics and Child Health, Faculty of Medicine and Health Sciences, Stellenbosch University, Cape Town, South Africa
2. Centre for Infectious Disease Epidemiology and Research, School of Public Health and Family Medicine, Faculty of Health Sciences, University of Cape Town
3. Health Impact Assessment Directorate, Western Cape Government, Department of Health
4. DSI-NRF South African Centre of Excellence in Epidemiological Modelling and Analysis (SACEMA), Stellenbosch University, Stellenbosch, South Africa

S1 Table. Initial loss to follow up TB patients, stratified by record of primary health care facility attendance, Cape Town, South Africa, October 2018-March 2020 (n=2,742)

		No recorded attendance at PHC		Attended PHC before TB diagnosis	
		441	col%	2,301	col%
Age category	0-4 years	37	*8.4*	265	*11.5*
	5-14 years	24	*5.4*	149	*6.5*
	15-24 years	32	*7.3*	222	*9.6*
	25-34 years	102	*23.1*	544	*23.6*
	35-44 years	92	*20.9*	456	*19.8*
	45-54 years	68	*15.4*	353	*15.3*
	55-64 years	48	*10.9*	200	*8.7*
	65+ years	38	*8.6*	112	*4.9*
Sex	Female	202	*45.8*	1,114	*48.4*
missing=4	Male	239	*54.2*	1,183	*51.4*
HIV status	HIV+ no ART	81	*18.4*	147	*6.4*
	HIV+ on ART	113	*25.6*	848	*36.9*
	HIV-	247	*56.0*	1,306	*56.8*
Diagnostic method	Clinically diagnosed	178	*40.4*	460	*20.0*
	Bacteriologically confirmed	263	*59.6*	1,841	*80.0*
Site of disease	EPTB	105	*23.8*	316	*13.7*
	PTB	3	*0.7*	275	*12.0*
	Site not specified	333	*75.5*	1,710	*74.3*
Diabetes	No	405	*91.8*	2,140	*93.0*
	Yes	36	*8.2*	161	*7.0*
Level of care where diagnosis was made	Primary Health Care	0	*0.0*	1,201	*52.2*
	District Hospital	140	*31.7*	255	*11.1*
	Regional Hospital	81	*18.4*	292	*12.7*
	Tertiary Hospital	220	*49.9*	553	*24.0*
TB treatment started	No	154	*34.9*	982	*42.7*
	Yes	287	*65.1*	1,319	*57.3*
Dead	No	246	*55.8*	2,028	*88.1*
	Yes	195	*44.2*	273	*11.9*
Timing of death	≤30 days	158	*35.8*	168	*7.3*
	>30 days	37	*8.4*	105	*4.6*

ART: Antiretroviral therapy; EPTB: extra pulmonary TB; PTB: pulmonary TB; TB: tuberculosis; +: positive; -: negative

S2 Table. Univariate and multivariable Cox regression model with predictors of death among TB patients who were initial loss to follow-up, Cape Town, South Africa, October 2018-March 2020 (n=2,742*)

		Total	Deaths	*CFR%*	HR (95%CI)	aHR (95%CI)
Age category	0-4 years	302	22	*7.3*	1.88 (0.64-5.58)	1.55 (0.58-4.09)
	5-14 years	173	5	*2.9*	reference	
	15-24 years	254	29	*11.4*	3.59 (1.24-10.36)	3.29 (1.27-8.55)
	25-34 years	646	94	*14.6*	5.43 (1.92-15.36)	3.01 (1.21-7.49)
	35-44 years	548	105	*19.2*	8.7 (2.96-25.55)	3.86 (1.55-9.62)
	45-54 years	421	79	*18.8*	11.22 (3.61-34.84)	3.33 (1.33-8.32)
	55-64 years	248	73	*29.4*	22.62 (6.87-74.5)	5.93 (2.38-14.79)
	65+ years	150	61	*40.7*	35.4 (10.03-125)	6.15 (2.46-15.37)
Sex	Male	1,422	231	*16.2*	reference	
	Female	1,316	237	*18.0*	1.15 (0.96-1.38)	1.11 (0.92-1.34)
HIV	HIV-	1,553	199	*12.8*	reference	
	HIV+ no ART	228	82	*36.0*	3.33 (2.57-4.32)	3.13 (2.36-4.15)
	HIV+ on ART	961	187	*19.5*	1.17 (0.95-1.42)	1.5 (1.18-1.91)
Diagnostic method	Bacteriologically confirmed	2,104	323	*15.4*	reference	
	Clinically diagnosed	638	145	*22.7*	1.91 (1.57-2.33)	1.59 (1.28-1.97)
Diabetes	No	2,545	412	*16.2*		
	Yes	197	56	*28.4*	1.51 (1.14-2.01)	0.92 (0.68-1.24)
Site of disease	PTB	278	3	*1.1*		
	EPTB	421	105	*24.9*	32.49 (10.31-102.39)	10.7 (3.37-34.04)
	Site not specified	2,043	360	*17.6*	48.66 (15.61-151.64)	14.64 (4.65-46.05)
Level of care of diagnosis**	Primary Health Care	1,201	68	*5.7*	reference	
	Hospital	1,541	400	*26.0*	4.94 (3.80-6.42)	
TB treatment started**	No	1,136	228	*20.1*	reference	
	Yes	1,606	240	*14.9*	0.13 (0.11-0.16)	
Level of care of diagnosis and treatment status	PHC diagnosis and started TB treatment	551	19	*3.4*	reference	
	PHC diagnosis not started on TB treatment	650	49	*7.5*	24.8 (14.47-42.48)	14.87 (8.64-25.58)
	Hospital diagnosis and started TB treatment	1,055	221	*20.9*	10 (6.26-15.99)	6.98 (4.3-11.32)
	Hospital diagnosis not started on TB treatment	486	179	*36.8*	58.42 (36.27-94.11)	34.56 (21.26-56.17)

ART: Antiretroviral therapy; aHR: adjusted hazard ratio; EPTB: extra pulmonary TB; HR: hazard ratio; PTB: pulmonary TB; TB: tuberculosis; +: positive; -: negative

*there were 2,742 TB patients who were initial loss to follow-up but 4 did not specify sex as male or female and 5 had insufficient dates for the calculation of person time. In the univariate analyses for sex and in the final multivariable model n=2,733; in all other univariate analyses n=2,737

** Variables not included in multivariable model due to collinearity. Level of care of diagnosis and TB treatment started collinear with *Level of care of diagnosis and treatment status*
